# A co-registration method to validate *in vivo* optical coherence tomography in the breast surgical cavity

**DOI:** 10.1016/j.heliyon.2024.e41265

**Published:** 2024-12-15

**Authors:** Rowan W. Sanderson, Renate Zilkens, Peijun Gong, Imogen Boman, Ken Y. Foo, Skandha Shanthakumar, James Stephenson, Wei Ling Ooi, José Cid Fernandez, Synn Lynn Chin, Lee Jackson, Mireille Hardie, Benjamin F. Dessauvagie, Anmol Rijhumal, Saud Hamza, Christobel M. Saunders, Brendan F. Kennedy

**Affiliations:** a BRITElab, Harry Perkins Institute of Medical Research, QEII Medical Centre Nedlands and Centre for Medical Research, The University of Western Australia, Perth, Australia; b Department of Electrical, Electronic and Computer Engineering, School of Engineering, The University of Western Australia, Perth, Australia; c Division of Surgery, Medical School, The University of Western Australia, Perth, Western Australia, Australia; d OncoRes Medical, Perth, Western Australia, Australia; e Breast Centre, Fiona Stanley Hospital, Murdoch, Western Australia, Australia; f PathWest, Fiona Stanley Hospital, 11 Robin Warren Drive, Murdoch, WA, 6150, Australia; g Division of Pathology and Laboratory Medicine, Medical School, The University of Western Australia, Perth, WA, 6009, Australia; h Clinipath Pathology, Suite 1, 302 Selby Street North, Osborne Park, WA, 6017, Australia; i Department of Surgery, Medical School, The University of Melbourne, Melbourne, Vic, Australia; j Institute of Physics, Faculty of Physics, Astronomy and Informatics, Nicolaus Copernicus University in Toruń, Grudziadzka 5, 87-100, Torun, Poland; k Australian Research Council Centre for Personalised Therapeutics Technologies, Melbourne, Australia

## Abstract

Breast-conserving surgery accompanied by adjuvant radiotherapy is the standard of care for patients with early-stage breast cancer. However, re-excision is reported in 20–30 % of cases, largely because of close or involved tumor margins in the specimen. Several intraoperative tumor margin assessment techniques have been proposed to overcome this issue, however, none have been widely adopted. Furthermore, tumor margin assessment of the excised specimen provides only an indirect indication of residual cancer in the patient following excision of the primary tumor. Handheld optical coherence tomography (OCT) probes and their functional extensions have the potential to detect residual cancer *in vivo* in the surgical cavity. Until now, validation of *in vivo* OCT has been achieved through correlation with *ex vivo* histology performed on the specimen removed during surgery that is adjacent to the tissue scanned *in vivo*. However, this indirect approach cannot accurately validate *in vivo* imaging performance. To address this, we present a method for robust co-registration of *in vivo* OCT scans with histology performed, not on the main specimen, but on cavity shavings corresponding directly to the tissue scanned *in vivo*. In this approach, we use *ex vivo* OCT scans as an intermediary, surgical sutures as fiducial markers, and extend the *in vivo* field-of-view to 15 × 15 mm^2^ by acquiring partially overlapping scans. We achieved successful co-registration of 78 % of 139 *in vivo* OCT scans from 16 patients. We present a detailed analysis of three cases, including a case where a functional extension of OCT, quantitative micro-elastography, was performed.

## Highlights

• *In vivo* imaging of the breast surgical cavity may improve the assessment of residual cancer.
• OCT scanning of breast tissue shavings provides a direct means of validating *in vivo* imaging.
• Co-registration between *in vivo* OCT and histology is improved by intermediary *ex vivo* OCT imaging.
• Tissue features and the proportion of adipose tissue impact co-registration accuracy.

## Graphical abstract

**Figure:**
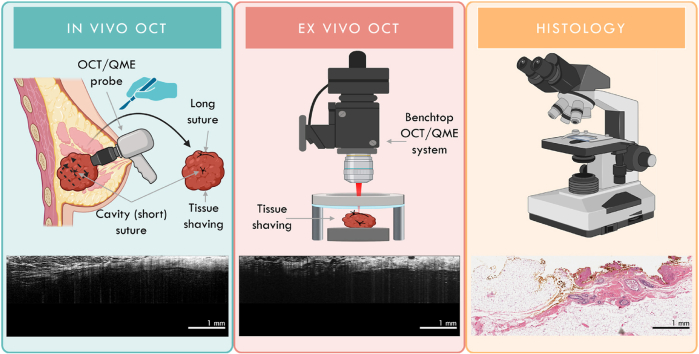

## 1 Introduction

Breast-conserving surgery (BCS) accompanied by adjuvant radiotherapy is the current standard of care for patients with early-stage breast cancer [1]. Overall survival and long-term disease-free survival are equivalent for patients undergoing either BCS with radiotherapy or mastectomy if clear tumor margins are obtained in the excised specimen [[2], [3], [4], [5]]. BCS also preserves cosmesis, which is linked to improved functional and psychological outcomes [6,7]. However, obtaining suitably clear margins remains a considerable challenge. Post-operative histology reveals close (<2 mm) or involved (0 mm) margins in 20–30 % of cases [8,9], which indicates the presence of residual cancer in the breast and increases the likelihood of local recurrence [10]. These patients are normally recommended for a repeat surgical procedure (*i.e.*, a re-excision), which can delay adjuvant therapies, leads some patients to choose mastectomy [11], increases morbidity and patient distress, results in poor cosmesis, and increases healthcare costs [12,13].

Close and involved margins are largely attributed to a lack of effective intraoperative tools, with surgeons often relying on tactile and visual information, which are both highly subjective [8]. Several diagnostic techniques have been proposed for intraoperative margin assessment, including frozen section histology [14,15], imprint cytology [16,17], and intraoperative specimen radiography [18]. However, these techniques have not been widely adopted by surgeons owing either to prohibitive time requirements, the need for pathologists to be present during the surgery, or low clinical efficacy [19]. Moreover, a main limitation of existing intraoperative assessment techniques is that they only examine the surgical margins of the specimen, which is not always an accurate reflection of residual cancer in the surgical cavity [20]. Additionally, it is challenging to relate tumor detected on the excised specimen to the precise corresponding location in the surgical cavity, which can result in the surgeon either missing the residual cancer or removing excess uninvolved tissue [21,22]. To address this, direct *in vivo* assessment of the surgical cavity following the primary excision may provide a more accurate assessment of residual cancer in the patient.

Optical coherence tomography (OCT) is a high-resolution optical imaging technique that has been proposed, in the form of handheld imaging probes, for *in vivo* assessment of cancer in the surgical cavity during breast-conserving surgery [[23], [24], [25], [26], [27]]. OCT provides spatial resolution of 5–15 μm and is capable of rapid volumetric acquisition with an imaging depth up to 1 mm in dense tissue [[28], [29], [30]]. OCT does not require contrast agents, fixatives, or tissue dissection, meaning it does not impact the diagnostic capability of postoperative histology. Furthermore, functional extensions of OCT such as polarization-sensitive OCT [31,32], OCT attenuation imaging [27,33], dynamic OCT [34], and quantitative micro-elastography (QME) [35], can generate additional contrast between benign and cancerous tissues, potentially enhancing the diagnostic accuracy [[36], [37], [38], [39]]. These combined capabilities make OCT and its functional extensions well-suited to *in vivo* detection of residual cancer.

So far, preliminary studies have been performed demonstrating the feasibility of *in vivo* OCT scanning of the surgical cavity. However, prior to performing large-scale clinical studies to determine diagnostic accuracy, it is necessary to first verify that the imaging performance observed *ex vivo* is maintained *in vivo*. This ensures optimal imaging performance and avoids propagating flaws in the imaging technique (*e.g.*, image artifacts) into the calculation of sensitivity and specificity. A main challenge is how to achieve accurate clinical validation of *in vivo* OCT scans of the surgical cavity. Until now, the approach has mainly been to correlate *in vivo* OCT scans with postoperative histology performed on the corresponding surface of the excised specimen [24]. However, such validation is inherently limited, as it is performed on adjacent tissue rather than on the exact tissue scanned *in vivo*. Additionally, given the malleability of excised breast tissue, maintenance of tissue orientation relative to the surgical cavity is very challenging, making correspondence between the *in vivo* OCT scans and histology difficult to achieve.

Here, we propose a methodology for co-registration of *in vivo* OCT breast scans with histology of the cavity shaving removed after OCT scanning that corresponds directly to the tissue scanned *in vivo*. Our method incorporates the use of intermediary *ex vivo* OCT imaging, fiducial markers, and a 3 × 3 grid of partially overlapping *in vivo* OCT scans to extend the imaging field-of-view to ∼15 × 15 mm^2^, enabling greater coverage of the surgical cavity than previously demonstrated [24,27]. We demonstrate our method using a handheld OCT probe to scan the surgical cavity of 16 BCS patients, following excision of the primary cancer, before co-registering the *in vivo* OCT images with *ex vivo* wide-field OCT images of the cavity shaving. We then co-register the *ex vivo* OCT images with histology of the shaving. Using this method, we achieved visual co-registration of 78 % of the 139 *in vivo* OCT scans with corresponding *ex vivo* OCT. We identified tissue microarchitecture in the *in vivo* OCT images with similar image contrast to the corresponding *ex vivo* images, including in veins, arteries, and nerves. Finally, we demonstrate how this technique can be extended to co-register functional extensions of OCT, namely QME.

## 2 Materials and methods

### 2.1 Study design

This study was performed at Fiona Stanley Hospital, Western Australia, with ethics approval from the South Metropolitan Health Service Human Research Ethics Committee (PRN: RGS0000000499 – approved on July 18, 2018). Adult female patients (*n* = 36) undergoing BCS were recruited with written informed consent acquired prior to surgery. Sixteen of these patients were scanned *in vivo* using the handheld OCT probe and had shavings taken during surgery (at the discretion of the operating surgeon) which were then scanned *ex vivo* with a bench-top OCT system. The remaining 20 patients were consented but excluded from the final analysis due to either no shaving being taken (*n* = 13), the shaving being taken prior to *in vivo* imaging (*n* = 4), the shaving being placed into formalin prior to *ex vivo* imaging (*n* = 1), orientation suture missing (*n* = 1), or technical problems with the imaging equipment prior to scanning (*n* = 1). This study was performed within the existing standard of care for BCS in accordance with the relevant guidelines and regulations, including following good clinical practice described at the International Conference on Harmonisation [40].

### 2.2 Clinical protocol

**Fig. 1:**
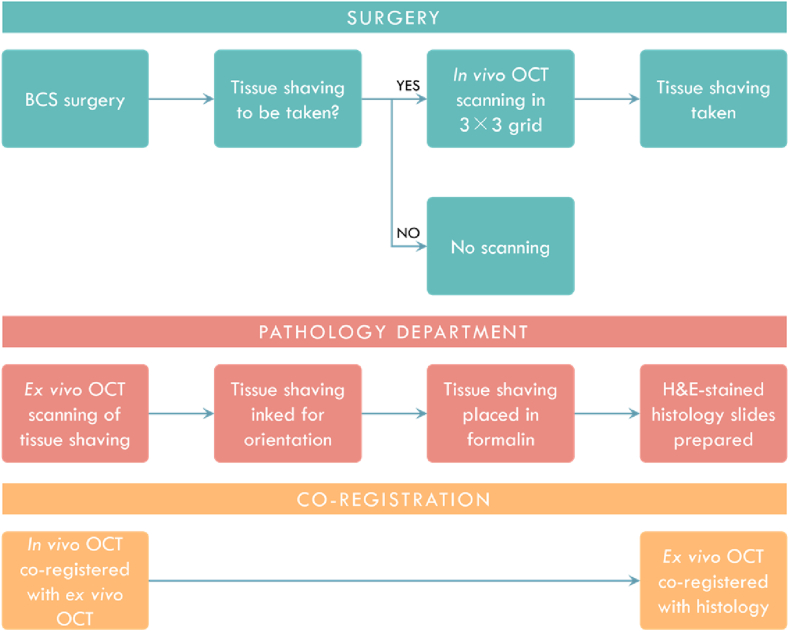
Clinical workflow of co-registration procedure.

Prior to surgery, the handheld OCT probe was sterilized with hydrogen peroxide plasma (STERRAD 100NX, Advanced Sterilization Products, Australia) by the hospital's central sterile services department. During sterilization, the cables and optical fiber connecting to the probe were protected against electromagnetic pulses within the sterilization chamber by stainless steel and copper shielding. This method of sterilization has been shown previously to have a negligible impact on the function and image quality of the OCT devices over multiple cycles [26,41]. At the beginning of the surgery, the probe was prepared in the sterile field by the theatre nurse. A sterilized disposable cap was placed on the distal end of the probe prior to imaging, which enabled QME [42], in addition to OCT. As the probe and cap had both been sterilized, there was no need to cover the probe with an additional sterile sheath as is commonly used with some medical devices. After excising the primary tumor, the surgeon assessed which area(s) of the surgical cavity required additional removal of breast tissue (*i.e.*, cavity shavings). If the surgeon suspected that residual cancer was present in the surgical cavity, they elected to take an additional cavity shaving of the corresponding cavity aspect. If a shaving was to be taken, the surgeon first performed *in vivo* OCT imaging by scanning with the handheld probe across the suspicious cavity aspect. Once scanning was complete, the surgeon returned the handheld probe to the theatre nurse and continued the surgery by performing a shaving of the cavity wall to remove additional tissue. To assist with orientation, a long suture was tied at the top of the excised shaving relative to the orientation of the acquired scans. The primary tumor and shavings were then transported immediately to the pathology department for bench-top OCT imaging of the shavings and routine postoperative histology. A summary of the clinical workflow is provided in Fig. 1.

### 2.3 *In vivo* optical coherence tomography

*In vivo* OCT imaging was performed using a custom handheld imaging probe connected to a spectral-domain OCT system (Telesto 320, Thorlabs, USA). The OCT system comprised a superluminescent diode source with a central wavelength of 1300-nm, a 3-dB spectral bandwidth of 170 nm and a line scan rate of 146 kHz. A fiber optic cable coupled the light from the OCT system to the probe where it was focused into the tissue, providing axial and lateral resolution of 5.5 μm and 14.1 μm, respectively (full width at half-maximum – in air). The OCT probe was arranged in a common-path configuration, with the distal end of the imaging window acting as the reference reflector. The probe used a disposable cap that contains a thin compliant silicone layer positioned between two thin membranes to facilitate QME imaging. Each *in vivo* OCT scan was acquired using a microelectromechanical system (MEMS) mirror to scan the beam across the tissue surface in a custom bidirectional scan pattern that enabled volumetric acquisition over 6 × 6 × 3.5 mm^3^ (474 × 474 × 1024 pixels) in 5.6 s [43]. The probe also contained an annular piezoelectric actuator which imparted microscale displacement to the tissue to facilitate QME scanning. The actuator was driven with a square wave by waveform generator (33500B Trueform Waveform Generator, Keysight Technologies, USA) and high voltage amplifier (PX200, PiezoDrive, Australia) and the deformation was synchronized with OCT acquisition, enabling OCT and QME data to be acquired simultaneously. The OCT system, waveform generator, high voltage amplifier, and controlling computer were housed on an imaging stack outside the surgical sterile field and were connected to the probe by a single bundled cable.

For *in vivo* scanning, the surgeon first tied a short suture in the cavity at the region where they suspected the residual cancer was located, following standard surgical protocol. The surgeon then positioned the probe ∼5 mm adjacent to the cavity suture and instructed the engineer, who controlled the acquisition software on the computer, to acquire a volumetric scan. The surgeon was then instructed by the clinical coordinator to move the probe to the next position by shifting it ∼5 mm either horizontally or vertically and to acquire another scan. The clinical coordinator recorded the location and order of each scan as well as any notes from the surgeon regarding the scan, *e.g*., poor contact between probe and tissue or excess blood in the cavity. This process was repeated until nine scans had been acquired, forming a 3 × 3 grid of scans, centered on the cavity suture. All scanning was completed in under 10 min and once scanning had finished, the surgeon returned the probe to the theatre nurse and continued the surgery by excising the shaving.

### 2.4 *Ex vivo* optical coherence tomography

The benchtop imaging system comprised an OCT system, an annular ring actuator, and wide-field translation stages. The *ex vivo* OCT system also used a Thorlabs Telesto III spectral-domain OCT system, which was connected to a standard scanner (OCTG13, Thorlabs, USA) with a focusing lens (LSM04, Thorlabs, USA) that provided axial and lateral resolutions of 5.5 μm and 13 μm, respectively (full width at half-maximum – in air). The system was configured in common-path with the light focused just below the imaging window, which was rigidly affixed to the 65 mm-diameter ring actuator (Piezomechanik GmbH, Germany). The ring actuator was connected to a waveform generator (33500B Trueform Waveform Generator, Keysight Technologies, USA) and high voltage amplifier (LE200/070, Piezomechanik GmbH, Germany) and imparted a 10 μm-displacement to the tissue synchronously with OCT acquisition. The signal used to trigger the scanning mirrors for B-scan acquisition was also used to trigger the ring actuator, ensuring that both were synchronized. The focusing lens provided a 16 × 16 mm^2^ lateral field-of-view, however, the tissue shavings were often larger than these dimensions. Therefore, to capture the whole tissue, the ring actuator, axial translation stage, and sample were translated by two lateral mechanical translation stages (*x*- and *y*-direction - NRT100, Thorlabs, USA) [44]. Scans were acquired at multiple locations and then stitched together to produce a 45 × 45 × 3.5 mm^3^ (2320 × 2320 × 1024 pixels) wide-field OCT scan of the tissue.

In the pathology department, the shaving was scanned using the benchtop OCT system. The tissue was placed on the base plate of a motorized axial translation stage (MLJ050, Thorlabs, USA) and the long suture was used to orientate the tissue shaving on the base plate relative to the *in vivo* OCT scan orientations (*i.e.*, long suture at the top of the *ex vivo* OCT image). A 500 μm-thick, 55 mm-diameter transparent silicone layer was placed on the tissue to enable stress measurements for QME [42]. The layer and sample were then brought into contact with the imaging window by the axial translation stage and pre-strained to ensure the shaving was in sufficient contact. *Ex vivo* wide-field OCT scanning was completed within 1 h of the tissue arriving at the pathology department and, once scanning was complete, the shaving was inked to orientate the *ex vivo* image relative to histology. After inking, routine histology of the shaving was prepared. Additional detail on the co-registration workflow and specimen orientation is provided in Supplementary Fig. S2.

### 2.5 Quantitative micro-elastography

In addition to OCT imaging, we also co-registered *in vivo* and *ex vivo* QME scans. QME has been described in detail previously [42]. Briefly, it is a functional extension of OCT that uses quasi-static compressive loading to map the elasticity of tissue in 3-D. In QME, a thin, pre-characterized silicone layer is positioned on top of the sample. During imaging, both the sample and layer are pre-compressed by an axial load. Microscale displacement is then applied to the layer and sample using a piezoelectric actuator, inducing local strain. The local strain in the layer is used to determine the corresponding local stress, which is assumed to be uniaxial. The ratio of axial stress to axial strain provides a measure of elasticity, which is determined at each voxel within the acquired image. Importantly, QME images are generated from the same data as that used to generate OCT images.

### 2.6 Postoperative histology

Postoperative histology was prepared using standard protocols for breast tissue specimens at PathWest, Fiona Stanley Hospital. The shavings were sectioned in a bread loafing style, resulting in several ∼4-5 mm-thick sections. The tissue sections were then fixed in 10 % neutral buffered formalin before being embedded in paraffin wax. A microtome then cut 4 μm slices from the block into a water bath, before the thin slices were transferred onto a glass coverslip. Excess paraffin wax was removed, and the slides were dried for several hours before the tissue was stained with hematoxylin and eosin (H&E). Finally, a glass coverslip was mounted over the tissue. Each histology slide was imaged with a digital microscope at × 20 magnification. The histology slides were annotated by an anatomical pathologist, who identified tissue structures and microarchitecture.

Importantly, the initial sectioning of the tissue was performed in the plane orthogonal to the *en face* plane of the acquired OCT volumes, following the standard protocol used in the hospital for shavings of breast margins. The tissue is sectioned in this way to enable the pathologists to perform routine margin assessment of the tissue specimen after imaging has been completed. This means that the subsequent histology slides were matched to the cross-sectional B-scan view in the corresponding OCT images, rather than the *en face* plane.

### 2.7 Image co-registration

To obtain reliable co-registration between *in vivo* OCT images and the corresponding histology of the cavity shaving, we first co-registered the *in vivo* OCT images, acquired with the probe, with the *ex vivo* OCT images, acquired with the benchtop system. To achieve this, the cavity suture was first identified in the *en face* plane of the *ex vivo* OCT volume, 100 μm below the tissue surface. The cavity suture at this position provided a distinct feature that was clearly visible in both *in vivo* and *ex vivo* OCT images. The scan that corresponded to this location was determined from the recorded clinical notes. By manually scrolling through the *en face* planes corresponding to different depths in the tissue, we identified which plane corresponded most closely to the cavity suture position and surrounding tissue appearance, considering in-plane angular rotation caused by slight differences in the angle of the *in vivo* OCT scans to the *ex vivo* OCT scan. Having co-registered the first *in vivo* OCT volume with the *ex vivo* OCT volume, subsequent *in vivo* OCT volumes were coarsely co-registered with the *ex vivo* OCT volume by approximately positioning them in the 3 × 3 grid, according to the scan locations recorded during surgery. Fine adjustments were then made by first looking for overlapping regions with the *in vivo* OCT volume at the cavity suture. As the *in vivo xy*-field-of-view is 6 × 6 mm^2^ and the surgeons were instructed to move ∼5 mm, there should be a ∼1 mm overlap between each *in vivo* volume. As highly precise motion on the order of 1 mm was difficult to achieve when imaging within a cavity, some scans overlapped by more than 1 mm, while others did not overlap at all. After looking for overlapping regions, *in vivo* OCT volumes were precisely co-registered by comparing visual features in the tissue (distinct adipose tissue and dense tissue regions) with the *ex vivo* OCT image. Using the same method of manually scrolling through the different *en face* planes, each volume was precisely co-registered with the *ex vivo* OCT volume.

In the second stage of co-registration, the *ex vivo* OCT volume was co-registered with the gold-standard H&E-stained histology images using a similar method to one developed previously [37]. Firstly, the histology grid, which represented the lines along which the tissue was initially sectioned, was overlaid on the wide-field *en face* OCT image, using the inked margins for orientation (see Supplementary Note S1). These lines approximate the locations from which the histology slides were taken. A correction factor was applied to the dimensions of the histology images to account for the shrinkage that was introduced during the histology process. This correction factor was determined by comparing the width of the fresh tissue with the width in the corresponding histology at the same location and was calculated for each histology slide such that each shaving had multiple correction factors to accommodate the variability of shrinkage within a shaving. The corresponding B-scans were then selected by analyzing a subset of B-scans located near the histology line and identifying the B-scans which had the strongest correspondence to the histology image, based on visual inspection.

## 3 Results

### 3.1 Optical coherence tomography co-registration

**Fig. 2:**
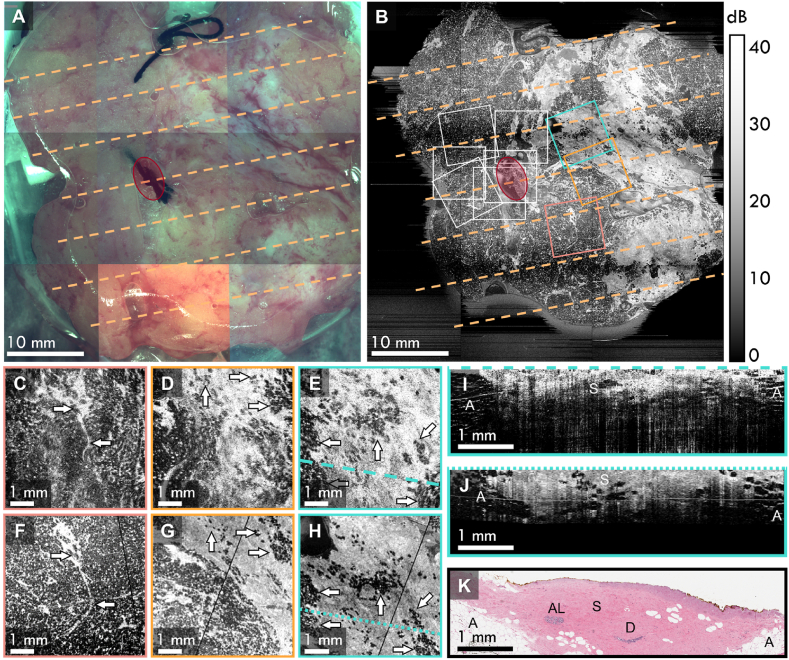
Co-registration of nine *in vivo* OCT scans with postoperative histology using *ex vivo* OCT as an intermediary. **A** Photograph of the tissue shaving obtained during *ex vivo* imaging and **B** the corresponding *ex vivo en face* OCT image of the specimen, with the *in vivo* scan locations represented by boxes. The dashed lines in **A** and **B** represent the locations of the histology sections and the red overlays highlight the short cavity suture. **C**, **D**, and **E** show select *en face* OCT images of *in vivo* scans compared to the corresponding *en face* images from the *ex vivo* OCT in **F**, **G**, and **H**, respectively. The white arrows depict features that are common in both the *in vivo* and *ex vivo* images. **I** The *in vivo* OCT B-scan corresponding to the long dashed line in **E**, **J** the co-registered *ex vivo* OCT B-scan corresponding to the short dashed line in **H**, and **K** the corresponding histology section. A – adipose tissue; AL – atrophic lobules; D – duct; S – stroma. All *en face* images are presented at 100 μm below the tissue surface.

Co-registration of *in vivo* OCT scans was performed for 16 cases. Here, we present detailed analysis of the co-registration between *in vivo* OCT and histology of three shavings that are representative of all cases in this study. The shaving in Fig. 2 is from a 71-year-old female patient undergoing BCS to remove a 46-mm invasive lobular carcinoma (ILC) with pleomorphic lobular carcinoma *in situ* (pLCIS) and ductal carcinoma *in situ* (DCIS) in the right breast. Following the cancer excision, an additional shaving was taken from the superior margin, shown in the photograph in Fig. 2A (breast shaving orientation is illustrated in Supplementary Fig. S1). Postoperative histology revealed regions of pLCIS and DCIS present within 1 mm of the superior margin of the shaving. The corresponding wide-field *en face* OCT image of the shaving at a depth of 100 μm below the tissue surface is shown in Fig. 2B, with the approximate locations of the histology blocks shown by the orange dashed lines, and co-registered locations of the nine *in vivo* OCT scans represented by the overlaid boxes. The position of the cavity suture is shown by the red overlay. Fig. 2B shows a mix of features (specifically, contrast between adipose tissue and dense tissue) and overlap between *ex vivo* and *in vivo* fields-of-view, which assist with the co-registration process.

The *en face* planes of three *in vivo* OCT scans are shown in Fig. 2C-E, along with the corresponding *en face* planes taken from the wide-field OCT image in Fig. 2F-H. The white arrows highlight features within the tissue that are common to both the *in vivo* and *ex vivo* images. There is a strong correspondence between the *in vivo* and *ex vivo en face* images, demonstrating that the *in vivo* OCT scans have been accurately co-registered with the wide-field OCT image. Slight differences in appearance are due to a combination of different spatial resolution of the two OCT systems, the presence of residual blood in the *in vivo* images, and small increases to the tilt of the probe, as seen between Fig. 2D and G. Nonetheless, these differences do not adversely impact the ability to co-register the two sets of images.

The next step requires co-registering the OCT scans with histology, which is prepared in the plane corresponding to the OCT B-scans. Fig. 2I shows the B-scan from an *in vivo* OCT scan corresponding to the dashed line in Fig. 2E, along with the corresponding B-scan from the wide-field OCT image in Fig. 2J. The OCT B-scans both show adipose tissue at the left and right edges, characterized by the low optical backscattering within the adipose cells, giving it a characteristic ‘honeycomb’-like appearance. The central regions of the B-scans show mostly benign dense stroma, characterized by high OCT intensity, with several adipose cells present throughout, corresponding well with the co-registered histology in Fig. 2K. The histology image does show the presence of atrophic lobules and a benign duct at a depth of ∼1 mm that are not visible in the OCT images. However, this is likely due to the dense stroma above these features heavily attenuating the OCT intensity. It is worth noting that the locations of the B-scans, denoted by the long and short dashed lines in Fig. 2E and H, do not correspond exactly to the location of the intersecting histology line in Fig. 2B. This is the result of the distortion and warping introduced by the histology preparation process compared to the fresh tissue. However, by adopting the method of scrolling through all neighboring B-scans to find the best correspondence, we are still able to co-register the images.

**Fig. 3:**
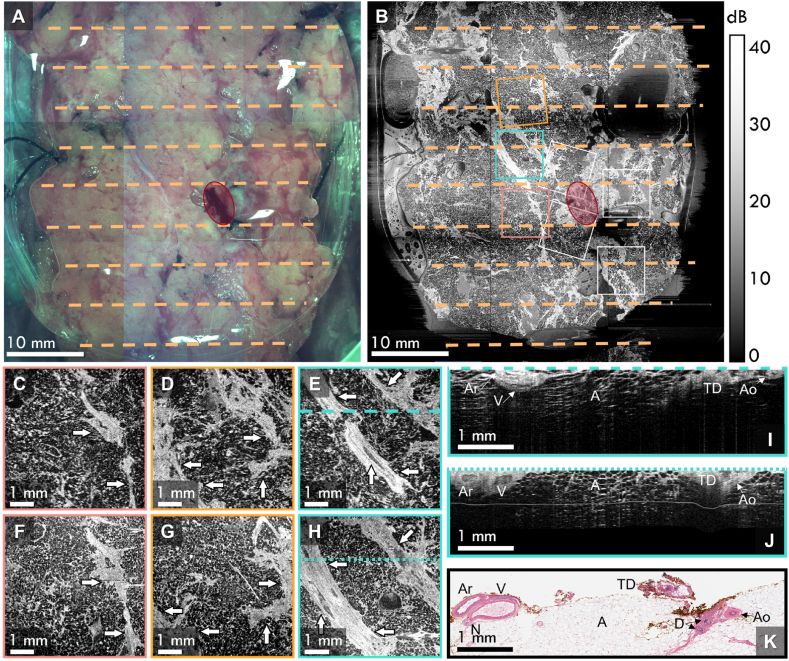
Co-registration of seven *in vivo* OCT scans with postoperative histology using *ex vivo* OCT as an intermediary. **A** Photograph of the tissue shaving obtained during the *ex vivo* imaging and **B** the corresponding *ex vivo en face* OCT image of the specimen, with the *in vivo* scan locations represented by the boxes. The dashed lines in **A** and **B** represent the locations of the histology sections and the red overlays highlight the short cavity suture. **C**, **D**, and **E** show select *in vivo en face* OCT images compared to the corresponding *ex vivo en face* OCT images in **F**, **G**, and **H**, respectively. The white arrows depict tissue microarchitecture that are captured in both the *in vivo* and *ex vivo* images. **I** The *in vivo* OCT B-scan corresponding to the long dashed line in **E**, **J** the co-registered *ex vivo* OCT B-scan corresponding to the short dashed line in **H**, and **K** the corresponding histology section. A – adipose tissue; Ar – artery; Ao – arteriole; D – duct; N – nerve; TD – thermal-damaged tissue; V – vein. All *en face* images are presented at 100 μm below the tissue surface.

Fig. 3 shows results from a 61-year-old female patient with a 12-mm mucinous carcinoma with DCIS in the right breast. Following cancer excision, an additional shaving was taken of the superior margin, shown by the photograph in Fig. 3A. The margins of the shaving were clear of invasive cancer. The wide-field *en face* OCT image of the shaving at a depth of 100 μm in the tissue is shown in Fig. 3B. Again, the histology grid and *in vivo* OCT scan locations are overlaid on the wide-field OCT image, represented by the dashed orange lines and the boxes, respectively. Select *in vivo en face* OCT scans are shown in Fig. 3C-E, along with the corresponding wide-field *en face* OCT scans in Fig. 3F-H. The white arrows highlight features in the tissue which are common in both sets of images. Similar to the results presented in Fig. 2C-H, there is good correspondence between the *in vivo* and *ex vivo en face* OCT images.

Fig. 3I and J shows *in vivo* and *ex vivo* OCT B-scans, respectively, corresponding to the long and short dashed lines in Fig. 3E and H. The co-registered histology is shown in Fig. 3K. On the left of the histology image, there is a neurovascular bundle consisting of an artery, a vein, and a nerve, which is also captured in both OCT B-scans. The artery and vein in the *in vivo* OCT B-scan are more compressed than in the *ex vivo* case, likely due to greater compressive force being applied by the probe in the cavity. Tissue thermally damaged by diathermy is shown to the right of all three images, which appears as a region of high OCT intensity in the B-scans. Finally, there is a small arteriole to the right of the thermally damaged tissue which appears as a ring in the OCT images.

In this case, only seven of the nine *in vivo* OCT scans could be co-registered with the wide-field OCT image, as two of the *in vivo* OCT scans were taken from regions on the tissue that correspond to an imaging artefact in the wide-field image. Such artifacts arise when the shavings exhibit substantial variations in surface topography causing local minima in the tissue surface to appear outside the *ex vivo* OCT imaging depth.

**Fig. 4:**
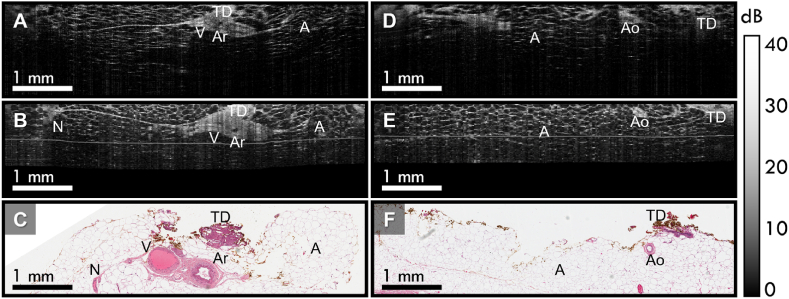
Breast tissue microarchitecture captured with *in vivo* and *ex vivo* OCT. **A***In vivo* OCT of a vein and artery, **B** the corresponding *ex vivo* OCT image, and **C** the corresponding histology section. **D***In vivo* OCT of an arteriole, **E** the corresponding *ex vivo* OCT image, and **F** the corresponding histology section. A – adipose; Ar – artery; Ao – arteriole; N – nerve; TD – thermal-damaged tissue; V – vein.

In addition to the co-registration shown in Fig. 3, we present two additional B-scan pairs with co-registered histology from this case in Fig. 4, highlighting the microarchitecture in the tissue. Fig. 4A, shows an *in vivo* B-scan containing a vein and artery, surrounded by a region of mostly adipose tissue. The same structures can clearly be seen in the corresponding *ex vivo* B-scan (Fig. 4B), with the addition of a nerve, and both B-scans correlate well with the histology in Fig. 4C. Fig. 4D shows a ∼180 μm-diameter arteriole surrounded by adipose tissue in an *in vivo* B-scan, validated by the co-registered histology in Fig. 4F. The same feature is also seen in the corresponding *ex vivo* B-scan in Fig. 4E. The cases presented in Fig. 4 highlight two cases where we were able to co-register small tissue features in regions that are predominantly adipose tissue, emphasizing the robustness of the co-registration method.

We consider the co-registration of *in vivo* to *ex vivo* OCT to be the main obstacle to reliable co-registration for *in vivo* OCT scans. Therefore, in Table 1, we summarize the *in vivo* to *ex vivo* co-registration success for all 16 cases based on whether the *in vivo* OCT scan contained mostly dense tissue, mostly adipose tissue or a mix of both. Success was determined by having two researchers independently attribute an *in vivo* OCT scan to the same region of the *ex vivo* OCT scan based on image appearance, proximity to the suture, and the location within the 3 × 3 grid of scans. A scan was considered to contain either mostly adipose tissue or dense tissue if more than 90 % of the field-of-view consisted of that tissue type.

**Table 1:** Co-registration success of *in vivo* OCT scans with *ex vivo* OCT scans based on tissue type.

	Tissue types scanned	Tissue types co-registered
	Number (Percentage of total data set)	Number (Success rate)
**Dense tissue**	11 (8)	10 (91)
**Adipose tissue**	31 (22)	15 (48)
**Mixed tissue**	97 (70)	84 (79)
**All**	**139 (100)**	**109 (78)**

From Table 1, we can see that the overall co-registration success rate was 78 %, and that is largely due to most scans containing a mix of tissue types. For scans containing mostly adipose tissue, 48 % were successfully co-registered. This reduction is due to a lack of discernible tissue features in the OCT images, unlike the examples shown in Fig. 4. Regions of mostly adipose tissue appear similar and there is a lack of certainty over the precise location of these scans relative to the wide-field image. Although the co-registration success rate for adipose tissue was lower than mixed tissue or dense tissue, it still allows for nearly half of the scans to be co-registered, despite the lack of identifying features. While regions of mostly dense tissue may also appear relatively similar, upon close examination there are often subtle variations in OCT intensity in these tissues, which are noticeable in both the *in vivo* and *ex vivo* images, making them easier to co-register. From this analysis, we can conclude that the co-registration technique works well when dense tissue is present, however, accuracy of adipose tissue regions may be improved by additional fiducial markers to provide additional features within the images. Another factor that reduces the co-registration accuracy is acquisition of *in vivo* OCT scans at the edge of the tissue subsequently removed as a shaving. In this case, the *in vivo* OCT scan often captured tissue that is not then excised, and therefore could not be co-registered with the wide-field image.

### 3.2 Quantitative micro-elastography co-registration

**Fig. 5:**
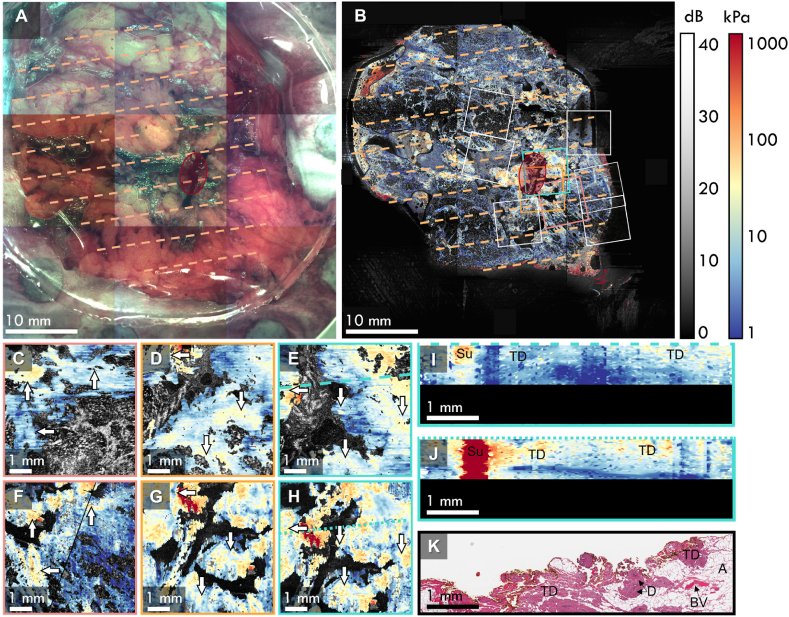
Co-registration of nine *in vivo* QME scans with postoperative histology using corresponding OCT. **A** Photograph of the tissue shaving obtained during *ex vivo* QME imaging and **B** the corresponding *ex vivo* QME image of the specimen, with the *in vivo* scan locations represented by the boxes. The dashed lines in **A** and **B** represent the locations of the histology sections and the red overlays highlight the cavity suture. **C**, **D**, and **E** show *in vivo en face* QME images compared to the corresponding *ex vivo en face* QME images in **F**, **G**, and **H**, respectively. The white arrows depict features that are common in both the *in vivo* and *ex vivo* QME images. **I** The *in vivo* QME B-scan corresponding to the long dashed line in **E**, **J** the co-registered *ex vivo* QME B-scan corresponding to the short dashed line in **H**, and **K** the corresponding histology section. The bottom regions of **I** and **J** have been masked out to remove regions of low elasticity sensitivity. A – adipose tissue; BV – blood vessel; D – ducts; Su – Suture; TD – thermal damaged tissue. All *en face* images are presented at 100 μm below the tissue surface.

In Fig. 5, we present co-registered QME scans acquired on a shaving taken from a 68-year-old female patient with a 12-mm invasive ductal carcinoma (IDC) in the left breast. The shaving was taken from the medial margin and was clear of invasive cancer. Co-registration for this case was performed on the OCT images acquired of the shaving and was then extended to the corresponding QME data. The photograph of the whole shaving is presented in Fig. 5A. The corresponding *en face ex vivo* QME image is shown in Fig. 5B, with the overlaid histology grid represented by the dashed orange lines and the *in vivo* QME scan locations represented by the boxes. The QME image presents the elasticity across the shaving on a logarithmic scale, which is overlaid on the corresponding OCT image. An automated segmentation algorithm was applied to the QME image, to remove regions of adipose tissue, as the low OCT intensity in adipose tissue leads to elasticity artifacts, and regions of non-contact [35].

*En face* images from three *in vivo* QME scans are shown in Fig. 5C-E. The corresponding wide-field QME images are shown in Fig. 5F-H, respectively, with the white arrows denoting features that are present in both the *in vivo* and *ex vivo* cases. Once again, there is a good correspondence between the tissue features present in both the *in vivo* and *ex vivo* QME images in addition to similar values of elasticity.

Fig. 5I shows an *in vivo* QME B-scan corresponding to the long, dashed line in Fig. 5E. The co-registered *ex vivo* QME B-scan is shown in Fig. 5J, corresponding to the short dashed line in Fig. 5H and the co-registered histology image is presented in Fig. 5K. The annotated histology shows substantial amounts of dense tissue that was thermally damaged by surgical diathermy. The elasticity shown in the QME images for these regions is ∼15–25 kPa, appearing slightly elevated compared to healthy dense tissue, which is consistent with previous reports on the effects of thermal damage on soft tissue [45].

## 4 Discussion

In this paper, we have described a method to co-register *in vivo* OCT images of tissue within the breast surgical cavity with histology of the corresponding cavity shaving. This method overcomes the main challenge with validating *in vivo* OCT scans by correlating histology on excised shavings corresponding directly to the tissue scanned *in vivo* with OCT. Our method achieves accurate co-registration by combining intermediary wide-field *ex vivo* OCT imaging, fiducial markers to aid with co-registration between *in vivo* and *ex vivo* OCT images, and acquisition of a grid of nine partially overlapping *in vivo* OCT scans that extend the effective field-of-view and enable the co-registration of scans in the absence of visual features. The benefit of first performing co-registration between histology of the shaving and *ex vivo* OCT of the shaving is that the tissue is imaged in a similar orientation (excluding the effects of shrinking and warping introduced during histology preparation), but with image contrast similar to *in vivo* imaging. This intermediate step makes it easier to map changes in tissue orientation. Additionally, using surgical sutures as fiducial markers that were visible in both the *in vivo* and *ex vivo* OCT scans provides a central reference point that is used to reliably co-register the first *in vivo* OCT scan to the wide-field image. Finally, co-registration of the remaining scans is achieved by the surgeon scanning in a known grid of partially overlapped scans, increasing the effective *in vivo* field-of-view. This also allows for scans to be co-registered relative to one another, in the absence of visible features in the tissue, and subsequently co-registered with the *ex vivo* image, via the *in vivo* OCT scan of the cavity suture. The benefit of this approach is that if one technique is unable to achieve co-registration, others can be used to compensate, making it more robust.

This method has several limitations. While the cavity suture provided a reliable marker for co-registration, it also impacted image quality. As the suture protrudes above the tissue surface, the tissue nearest the suture is occasionally not captured in the OCT scans. This creates a problem as the surgeon ties the cavity suture at the region that they believe is most likely to contain cancerous tissue. This procedure was part of the established clinical protocol and thus, could not be changed for this study. However, in future studies, the use of indelible ink imaged with a camera co-aligned to the OCT field-of-view could be used instead of the suture to overcome this issue. Indelible ink would provide a common landmark in both the *in vivo* and *ex vivo* images without affecting the contact at the surface, enabling more tissue to be imaged by OCT.

Another limitation of this approach is that it only considers rotation of the *xy*-plane (*i.e.*, yaw). The method does not account for tilt of the probe relative to the tissue surface for *in vivo* OCT scans. Therefore, when comparing the *en face* planes, regions of the *in vivo* OCT image may not match the corresponding wide-field OCT image, as it may have been acquired from a different depth in the tissue. Further compounding this issue is the nonlinear geometric distortion of the tissue during histology preparation. While these issues make it challenging to achieve a one-to-one co-registration on the micro-scale, we believe that the results shown here demonstrate a sufficiently high accuracy and precision which would enable future validation studies.

Finally, while we were able to achieve accurate co-registration with histology, the coverage of the *in vivo* OCT scans was ∼30–40 % of the total cavity shavings, as seen in Fig. 2, Fig. 3, Fig. 5B. In this study, the primary goal was to develop a methodology for accurate co-registration for the purpose of validating image performance and as such, we prioritized precise placement and overlapping of the nine scans acquired *in vivo* over complete shaving coverage. Due to the short acquisition time of 5.6 s and 10-min timeframe in which to complete the *in vivo* scanning, it is feasible in future studies to acquire substantially more *in vivo* OCT scans which would provide a more complete coverage of the shaving.

## 5 Conclusion

In this paper, we have presented a robust method for co-registration of *in vivo* OCT and QME images acquired in the surgical cavity during breast-conserving surgery to postoperative histology of corresponding cavity shavings, which fits within the existing clinical workflow and does not impact patient standard of care. We implemented this method on 16 patients and presented results from three representative cases, showing strong correspondence between the different images. We demonstrated successful co-registration of 109 of 139 (78 %) of *in vivo* OCT scans. We believe that our method provides a reliable means to validate the imaging performance of *in vivo* OCT imaging of the surgical cavity in BCS.

## CRediT authorship contribution statement

**Rowan W. Sanderson:** Writing – review & editing, Writing – original draft, Visualization, Validation, Project administration, Methodology, Investigation, Data curation, Conceptualization. **Renate Zilkens:** Writing – review & editing, Validation, Methodology, Data curation, Conceptualization. **Peijun Gong:** Writing – review & editing, Validation, Methodology, Data curation, Conceptualization. **Imogen Boman:** Writing – review & editing, Investigation, Data curation, Conceptualization. **Ken Y. Foo:** Resources, Investigation. **Skandha Shanthakumar:** Resources. **James Stephenson:** Resources, Investigation. **Wei Ling Ooi:** Resources, Investigation. **Jose Cid Fernandez:** Resources, Investigation. **Synn Lynn Chin:** Resources, Investigation. **Lee Jackson:** Resources, Investigation. **Mireille Hardie:** Resources, Investigation. **Benjamin F. Dessauvagie:** Resources, Investigation. **Anmol Rijhumal:** Resources, Investigation. **Saud Hamza:** Resources, Investigation. **Christobel M. Saunders:** Writing – review & editing, Resources. **Brendan F. Kennedy:** Writing – review & editing, Supervision, Resources, Project administration, Methodology, Funding acquisition, Conceptualization.

## Ethics approval statement

This study was approved by the South Metropolitan Health Service Human Research Ethics Committee (PRN: RGS0000000499 – approved on July 18, 2018). Written informed consent was acquired from all patients prior to their surgery and the study was carried out in accordance with the relevant guidelines and regulations, including following good clinical practice described at the International Conference on Harmonisation.

## Data availability statement

The data that support the findings of this study are available from the corresponding author upon reasonable request.

## Declaration of competing interest

The authors declare the following financial interests/personal relationships which may be considered as potential competing interests: Brendan F. Kennedy reports financial support was provided by OncoRes Medical. Brendan F. Kennedy reports financial support was provided by 10.13039/501100000923Australian Research Council. Brendan F. Kennedy reports financial support was provided by 10.13039/100031198Government of Western Australia Department of Health. Imogen Boman reports a relationship with OncoRes Medical that includes: employment. Skandha Shanthakumar reports a relationship with OncoRes Medical that includes: employment. Brendan F. Kennedy reports a relationship with OncoRes Medical that includes: equity or stocks. Brendan F. Kennedy has a patent for a device and a method for evaluating a mechanical property of a material Intl. Pub. No. WO/2016/ 119011, Appl. No. PCT/AU2016/212695, Priority date: 30/01/2015 issued to OncoRes Medical. Christobel M. Saunders is the Chief Medical Officer (not remunerated) for OncoRes Medical. The other authors declare that they have no known competing financial interests or personal relationships that could have appeared to influence the work reported in this paper.
