# Supplementary material for: A co-registration method to validate in vivo optical coherence tomography in the breast surgical cavity
Source: Heliyon. 2024 Dec 15;11(1):e41265. doi: 10.1016/j.heliyon.2024.e41265 (PMC11728906; doi:10.1016/j.heliyon.2024.e41265)
Supplement: Multimedia component 1 [file mmc1.docx]

**Supplementary information**

**A co-registration method to validate *in vivo* optical coherence tomography in the breast surgical cavity**

**Authors**

Rowan W. Sanderson,^1,2,*^ Renate Zilkens,^1,3^ Peijun Gong,^1,2^ Imogen Boman,^1,2,4^ Ken Y. Foo,^1,2^ Skandha Shanthakumar,^4^ James Stephenson,^5^ Wei Ling Ooi,^5^ Jose Cid Fernandez,^5^ Synn Lynn Chin,^5^ Lee Jackson,^5^ Mireille Hardie,^6^ Benjamin F. Dessauvagie,^6,7,8^ Anmol Rijhumal,^6^ Saud Hamza,^5^ Christobel M. Saunders,^3,9^ and Brendan F. Kennedy^1,2,10,11^

^1^BRITElab, Harry Perkins Institute of Medical Research, QEII Medical Centre Nedlands and Centre for Medical Research, The University of Western Australia, Perth, Australia

^2^Department of Electrical, Electronic and Computer Engineering, School of Engineering, The University of Western Australia, Perth, Australia

^3^Division of Surgery, Medical School, The University of Western Australia, Perth, Western Australia, Australia.

^4^OncoRes Medical, Perth, Western Australia, Australia.

^5^Breast Centre, Fiona Stanley Hospital, Murdoch, Western Australia, Australia.

^6^PathWest, Fiona Stanley Hospital, 11 Robin Warren Drive, Murdoch, WA, 6150, Australia

^7^Division of Pathology and Laboratory Medicine, Medical School, The University of Western Australia, Perth, WA, 6009, Australia

^8^Current affiliation: Clinipath Pathology, Suite 1, 302 Selby Street North, Osborne Park, WA, 6017, Australia

^9^Department of Surgery, Medical School, The University of Melbourne, Melbourne, Vic, Australia.

^10^Institute of Physics, Faculty of Physics, Astronomy and Informatics, Nicolaus Copernicus University in Toruń, Grudziadzka 5, 87-100 Torun, Poland.

^11^Australian Research Council Centre for Personalised Therapeutics Technologies, Melbourne, Australia

*Corresponding author: [rowan.sanderson@uwa.edu.au](mailto:rowan.sanderson@uwa.edu.au)


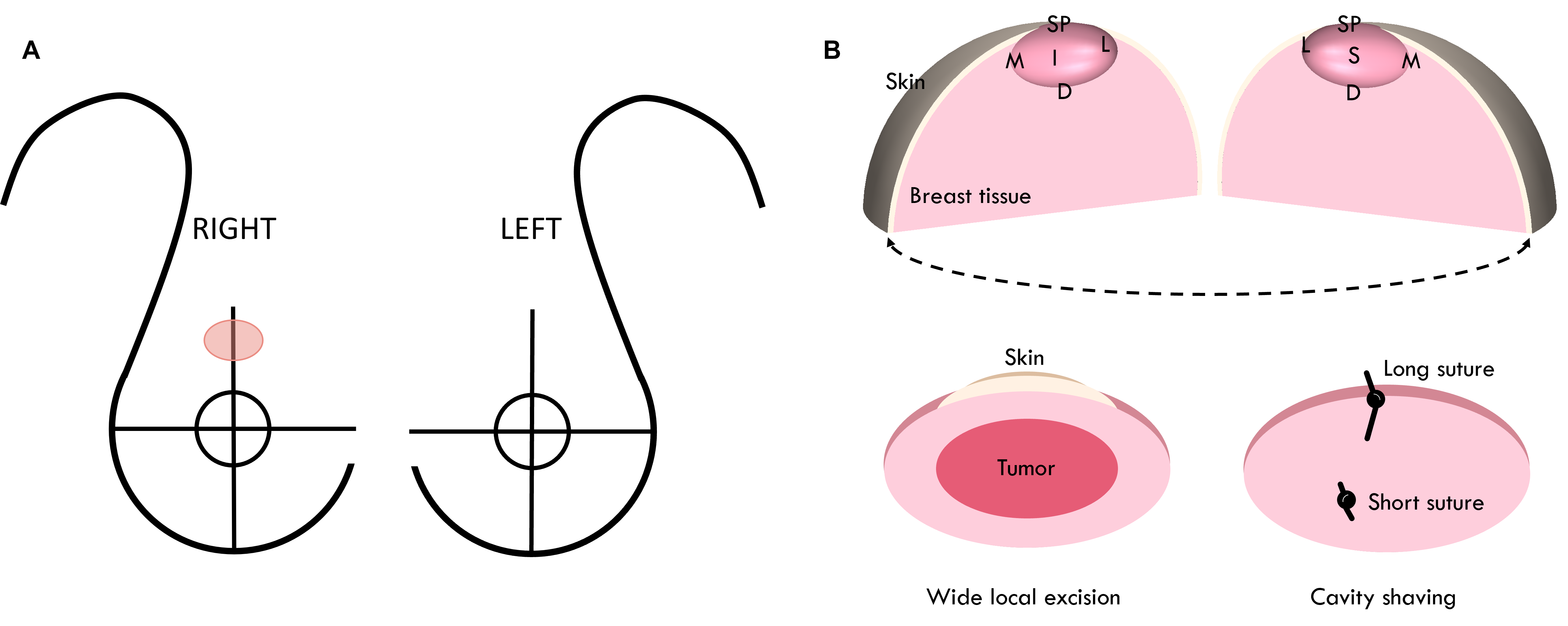


**Supplementary Figure S1:** Illustration of breast specimen orientation. **A** Representative diagram used to indicate from which breast the WLE was taken and the approximate location of the WLE in the breast. **B** Schematic of the cavity aspects in the right breast following WLE, including illustrations of a WLE specimen and a cavity shaving. D: deep; I: inferior; L: lateral; M: medial; S: superior; SP: superficial.

**Supplementary Figure S2:** Illustration of the imaging modalities and specimen orientations throughout the co-registration process of a shaving taken from the superior margin in the right breast of a 71-year-old female patient. **A** Photograph of the freshly excised breast shaving specimen showing the orientating long suture, which is tied at the superficial aspect and the short suture, which is tied on the cavity side of the shaving and indicates the suspected region of residual cancer. **B** *In vivo* OCT scans were acquired prior to excision, in a 3 × 3 grid, and **C** the same *in vivo* OCT scans arranged according to regions of overlap between adjacent scans. **D** Photograph of the breast shaving specimen taken while it was imaged with *ex vivo* OCT, **E** corresponding *ex vivo* widefield OCT scan, and **F** the *ex vivo* widefield OCT scan with the *in vivo* OCT scans in **C** overlaid. **G** Following *ex vivo* OCT scanning, the specimen was inked yellow at the cavity margin, black at the anti-cavity margin, blue at the lateral margin, and orange at the medial margin. After fixing the tissue in formalin, it was sectioned into multiple blocks according to the dashed lines. **H** Representative histology slide, stained with hematoxylin and eosin. The dashed boxes highlight regions of **I** blue ink, **J** black ink, and **K** yellow ink at the respective margins.

**Supplementary Note S1**

**Note on the projection of the histology grid onto the *ex vivo* OCT image**

The histology grid represents the approximate locations from where the main specimen was sectioned for histology preparation. To perform co-registration, this grid is projected onto the *ex vivo* OCT image so that the OCT B-scans can be co-registered with the corresponding histology slice. This projection is performed by initially numbering the histology slices according to the order in which they were cut. The spacing between each slice is ~5 mm, due to the dimensions of the cassettes that the slices are prepared within. Using the slice numbering and spacing, combined with the specimen inking, the location of each slice is approximated and subsequently projected onto the fresh tissue images to enable co-registration.
